# Intestinal microbiome-gut-brain axis and irritable bowel syndrome

**DOI:** 10.1007/s10354-017-0592-0

**Published:** 2017-09-08

**Authors:** Gabriele Moser, Camille Fournier, Johannes Peter

**Affiliations:** Division of Gastroenterology and Hepatology, Department of Internal Medicine III, Medical University of Vienna, Waehringer Guertel 18-20, Vienna, 1090 Austria

**Keywords:** Stress, Psyche, Depression, Anxiety, Enterotype, Stress, Psyche, Depression, Angststörung, Enterotyp

## Abstract

Psychological comorbidity is highly present in irritable bowel syndrome (IBS). Recent research points to a role of intestinal microbiota in visceral hypersensitivity, anxiety, and depression. Increased disease reactivity to psychological stress has been described too. A few clinical studies have attempted to identify features of dysbiosis in IBS. While animal studies revealed strong associations between stress and gut microbiota, studies in humans are rare. This review covers the most important studies on intestinal microbial correlates of psychological and clinical features in IBS, including stress, anxiety, and depression.

## Zusammenfassung

Psychische Komorbidität ist häufig bei Reizdarmsyndrom (RDS). Die aktuelle Forschung weist auf eine Rolle des intestinalen Mikrobioms bei der viszeralen Hypersensitivität, bei Angst und Depression hin. Eine Steigerung der Krankheitssymptomatik bei psychischem Stress wurde beschrieben. Wenige klinische Studien haben versucht, Muster einer Dysbiose für RDS zu identifizieren. Während Tierstudien eine starke Assoziation zwischen Stress und Darm-Mikrobiom aufdeckten, sind Studien beim Menschen rar. Diese Übersicht umfasst die wichtigsten Studien über Zusammenhänge zwischen dem intestinalen Mikrobiom und psychischen und klinischen Aspekten bei RDS, inklusive Stress, Angst und Depression.

## The intestinal microbiome, stress and the gut–brain axis

The human intestinal system is home to about 100 trillion microbes, mainly of bacterial origin [1]. Research has ascertained a critical relevance of gut bacteria for health and disease [2, 3], and there is strong evidence suggesting they can affect emotion processing and stress coping. The concept of a gut-brain-microbiota axis connects the psyche and nervous system with the intestine, its inhabitants and its metabolic, neuroendocrine, and immune functions [4, 5].

Gut microbiota sets developmental conditions for hypothalamic-pituitary-adrenal axis (HPA-axis) maturation [6, 7]. Together with an impact on brain circuits of social cognition, reward, and emotion processing, this indicates a relevance for resilience and behavioral adaption to stress [8, 9]. Vice versa, stress can decrease the diversity and alter the composition of the gut microbiome [10, 11]. Early life stress induced by maternal separation leads to lifelong alterations in microbial composition, HPA axis functioning and visceral hypersensitivity in rodents. This has been proposed as an animal model of irritable bowel syndrome (IBS) [12–14]. Loss of intestinal epithelial barrier function is another focus of research on the relationship between stress and microbiota. Suspected to provoke inflammatory cascades, autoimmunity, and pain, “leaky gut” can be both cause and consequence of psychological stress [15–17].

## Intestinal microbiome, inflammation and psychological disorders

Another strong link between intestinal microbiota and psychological health is based on the “inflammation hypothesis” of psychiatric disease [18, 19]. Bacterial pathogens can initiate inflammatory reactions [20] or modulate inflammatory processes via metabolites (e. g., short-chain fatty acids (SCFAs)) [21, 22]. Furthermore, gut bacteria are involved in the metabolism of key neurotransmitters such as serotonin [5], and seem to intervene in the turnover of neuronal growth factors related to cognition and brain health, e. g. brain-derived neurotrophic factor (BDNF) [23, 24]. Microbiota-dependent effects on mouse reward circuitry [25], and probiotics associated alterations of emotion-processing brain activity in humans [26] have been reported.

Behavioral alterations (anxiety and depression-like behavior) by manipulations of the microbiome were demonstrated in seminal animal studies [27–29]. Recently, socially avoidant behavior, paralleled by decreased myelinization in the prefrontal cortex, was ascribed to bacteria-mediated epigenetic modulations in mice [30]. In humans, Lin and colleagues [31] found significant differences in the composition of the gut microbiome between patients suffering from major depressive disorders and controls, displaying a dysbalanced Firmicutes to Bacteroidetes ratio and higher abundance of *Streptococcus*, *Klebsiella*, *Prevotella*, and *Clostridium XI* in depressive patients.

Those results contrast with the findings of Jiang and colleagues [32]. Although reporting the same higher Firmicutes to Bacteroidetes ratio, they observed different associations between taxa and major depressive disorder. *Faecalibacterium* was inversely correlated with symptoms severity, and an association between *Clostridium* cluster XIV and BDNF serum concentrations was observed.

In healthy women, however, no association between microbial features and psychological parameters could be found [33]. Further studies will have to determine the precise role of the microbiome within the range of normal behavior to pathophysiology in psychiatric illness.

## Irritable bowel syndrome and microbiome–brain interaction

Irritable bowel syndrome (IBS) is a functional gastrointestinal disorder with an estimated prevalence in the general population of 5 to 20% [34]. IBS is defined with following diagnostic criteria (ROME IV) [35]: recurrent abdominal pain, on average at least one day per week in the last 3 months. The pain should be related to defecation and associated with a change in frequency and in form (appearance) of stool.

IBS can be seen as a “stress disease” [36], and has been studied from different perspectives, at virtually all levels of the gut-brain-microbiome axis. There is evidence for HPA axis and autonomous nervous system involvement [37, 38], maladaptive coping and resilience [39–41], comorbidity of anxiety and depression [42], increased interoception [43] and altered neuronal pain processing [44, 45]. Host–microbe interactions in the gut are important elements in the pathogenesis of IBS and other functional gastrointestinal diseases [46].

A variety of methods have been used to assess the human intestinal microbiota in IBS and this might be one reason (along with different diets, lifestyles, and geographical factors) why results are contradictory and difficult to summarize. Many studies have attempted to identify microbiota discriminating IBS patients from healthy controls, and to define dysbiosis in IBS and its sub-populations [47, 48]. However, a widely accepted concept of bacterial dysbiosis in IBS has not yet been established, since a large proportion of IBS patients display a “normal-like” microbiota profile [49, 50]. Associations between clinical parameters and microbiota profiles assessed by classic ecological methods have remained largely elusive, but some bacteria stand out. A dysbalanced Firmicutes to Bacteroidetes ratio [49, 51], an increase in *Clostridium* XIVa and *Ruminococcus* [52], a reduction in *Bifidobacterium* [46, 52, 53], as well as a reduction in methanogens and butyrate- (a SCFA) producing bacteria in IBS with diarrhea (IBS-D) and IBS with diarrhea and constipation (mixed, IBS-M) patients [51, 54].

Some enterotypes and associations with clinical phenotypes have also been described: *Prevotella*-predominant enterotype seemed to be more common in healthy subjects, *Bacteroides* enterotype was more represented in IBS patients, while the *Clostridiales*-dominant enterotype was associated with faster colonic transit time. IBS severity could also be predicted by a complex microbial signature, consisting of bacterial families disseminated over the whole phylogenetic tree [54].

## Intestinal microbiota and psychological profiles in IBS

Jeffery and colleagues [49], comparing the fecal microbiota of IBS patients with non-IBS individuals, separated their IBS cohort into three main clusters: a “normal-like” cluster with microbial characteristics highly similar to healthy controls and two “IBS clusters,” the latter characterized by high Firmicutes to Bacteroidetes ratio. They also tested for associations between clinical variables and microbial composition, and found that depression was the single clinical feature that segregated in parallel with microbiota composition. Interestingly, depression was increased in patients belonging to the “normal-like” cluster.

Those findings are corroborated by Liu and colleagues [55], who observed that the fecal microbiome of IBS patients presented strong similarities to that of depressive patients. Sundin et al. [56] published data that distinguished the intestinal microbiota of post-infectious IBS patients from that of both general IBS patients and healthy controls. They also observed that altered fecal and mucosal microbial composition in post-infectious IBS patients correlated with psychological distress. Jeffery et al. [49] cautiously interpreted this pattern as more “physically-triggered” versus more “centrally-triggered” IBS. Our own data^1^ also strongly suggest that variables of psychological distress are associated with systematic microbial differences. Another recent study also found two clusters of IBS patients carrying either normal-like microbiomes or altered microbiomes. Here it was found that bacteria that carried metagenes involved in neurotransmitter metabolism correlated with morphological brain variations [57]. The pathways through which the gut microbiome affects the nervous system remain to be understood. However, some results point to western diet as a possible confounding factor: it affects the microbiota and its SCFA production, as well as the gut barrier integrity, and is associated with neuroinflammation and alteration of brain insulin sensitivity [58].

Therapeutic interventions are therefore possible from both ends of the spectrum: the brain and the gut microbiome. Antidepressants, psychotherapy and gut-directed hypnosis are effective, especially in refractory IBS [59, 60]. Until now, however, limited results are available from trials assessing the effectiveness of diet, prebiotics, probiotics, and antibiotics in IBS patients [61, 62].

## Conclusion

The gut microbiota forms a crucial link in the bidirectional interactions between the intestine and the nervous system. Some alterations, like psychological distress or gastrointestinal infections, can affect these interactions and contribute to the development and/or affect the course of IBS. Symptomatic therapies such as IBS-type related medication, phytopharmacology, diet, probiotics and psychological interventions including gut directed hypnosis are recommended.

Further randomized clinical trials are needed to identify those IBS patients who will profit more from a) therapies that modulate the gut microbiome (prebiotics, probiotics, antibiotics), b) psychological interventions (psychotherapy, gut-directed hypnosis, antidepressants), or c) both approaches within an integrated psychosomatic care.

## Footnotes

1 Abstract accepted for presentation at the United Gastroenterology Week 2017: Peter et al., Psychological distress is associated with gut microbial differences in Irritable Bowel Syndrome.
